# The Significance of Anti-Beta-2-Glycoprotein I Antibodies in Antiphospholipid Syndrome

**DOI:** 10.3390/antib5020016

**Published:** 2016-06-08

**Authors:** Anna Brusch

**Affiliations:** Department of Clinical Immunology, PathWest, Sir Charles Gairdner Hospital, Perth WA 6009, Australia; anna.brusch@health.wa.gov.au; Tel.: +61-8-9346-3333

**Keywords:** antiphospholipid syndrome, antiphospholipid antibody, beta-2 glycoprotein I, isotypes, domain specific antibodies

## Abstract

Antiphospholipid syndrome (APS) is a thrombophilic disorder that classically presents with vascular thrombosis and/or obstetric complications. APS is associated with antiphospholipid antibodies: a heterogeneous group of autoantibodies that are directed against membrane phospholipids in complex with phospholipid-binding proteins. Beta-2-glycoprotein I (B2GPI) binds anionic phospholipids and is considered to be the predominant antigen in APS and antibodies against B2GPI (anti-B2GPI) are recognised in the laboratory criteria for APS diagnosis. This review focuses on the part played by anti-B2GPI in the pathogenesis of APS, their associations with different clinical phenotypes of the disorder and new avenues for refining the diagnostic potential of anti-B2GPI testing.

## 1. Introduction

Antiphospholipid syndrome (APS) is an autoimmune thrombophilia that can occur in isolation or as part of an underlying systemic autoimmune disorder, such as systemic lupus erythematosus (SLE) [1]. It has a wide range of potential clinical manifestations: most commonly presenting with arterial or venous thrombosis, which may occur in the absence of other risk factors. Recurrent thrombosis can occur in some patients. Obstetric complications such as recurrent early miscarriages, preeclampsia and late fetal loss also form a subset of the disorder, known as obstetric APS [2]. While these clinical phenotypes are typical of APS, there are no pathognomic features that can secure the diagnosis of APS on clinical grounds alone. Rather, the diagnosis is made by the combination of clinical features together with supportive laboratory findings. This relies on the accurate identification and measurement of antiphospholipid antibodies (aPL). A number of aPL have been described in APS, however, only three are included in the current consensus guidelines regarding diagnosis [3]. These are lupus anticoagulant (LA), anticardiolipin antibodies (aCL) and anti-beta-2 glycoprotein I antibodies (anti-B2GPI). This review will focus on anti-B2PI and their role in APS in terms of their relationship to the putative pathogenesis of the disorder and their clinical associations. There are also areas of ongoing doubt such as the relative significance of particular anti-B2GPI isotypes and new areas of investigation including the potential for domain specific antibodies to refine the diagnostic value of anti-B2GPI testing.

## 2. Anti-B2GPI: Antibodies against an Enigmatic, Multi-Purpose Target

A role for anti-B2GPI in the pathogenesis of APS has been demonstrated in *in vivo* animal models [4]. It is hypothesised that anti-B2GPI bind to membrane-bound B2GPI complexed with anionic phospholipids expressed on the surface of a range of cells involved in the coagulation cascade which triggers cellular signaling events culminating in procoagulant effects such as modification of endothelial cells, potentiation of platelet aggregation and interference with plasma clotting components [5]. However, despite B2GPI being the predominant target in APS pathogenesis, its precise physiological function remains elusive.

The normal function of B2GPI has largely been inferred from scrutinising its complex protein structure. B2GPI contains five domains composed of repeating stretches of about 60 amino acids, similar to other proteins of the complement control protein superfamily [6]. The 5th domain contains a C-terminal extension and an additional disulphide bond that confers a positive charge resulting in an affinity for anionic phospholipids. The crystal structure of B2GPI was first elucidated in the late 1990s and demonstrated a stretched arrangement of domains 1–4, with the 5th domain protruding at a right angle giving an appearance resembling the letter ‘J’ or a hockey stick. Subsequent analysis by small angle X-ray scattering experiments suggested that in solution, B2GPI adopted an ‘S-shaped’ conformation [7]. More recently, electron microscopy studies also indicate that the structure of B2GPI is not limited to a single conformation. Rather, B2GPI can assume a different geometry in fluid phase which may alter its potential to interact with autoantibodies [8]. By electron microscopy analysis, B2GPI was found to assume a circular conformation in plasma with domains 1 and 5 opposed. In this form, the site(s) for autoantibody binding are shielded. Binding of anti-B2GPI to membrane-bound B2GPI stabilises its J-shaped structure and augments B2GPI’s interaction with membrane phospholipids which is hypothesised to potentiate B2GPI’s signaling through other transmembrane and intracellular ligands. These include toll-like receptors; TLR2 and TLR4, annexin A2 and LRP8 [6]. Signaling via these molecules mediates prothrombotic cellular actions.

In patients with APS, thrombotic events occur with increasing frequency in the presence of other prothrombotic risk factors such as infection. How these multiple ‘hits’ align to result in thrombosis is likely to be complex and multifactorial. However, recent studies have started to shed light on this area by indicating a potential interplay between B2GPI and various elements of the immune system during infection. For example, the positively charged sites in domain 5 of B2GPI confer an affinity for negatively charged cell membranes and are also thought to result in interactions with bacteria that might trigger innate immune responses. Indeed, peptides derived from domain 5 have been shown to display potent antibacterial activity against a variety of bacteria [9]. Other studies have shown that B2GPI interacts directly with lipopolysaccharide resulting in a complex that can be recognised and internalised by macrophages [10]. In this way, B2GPI’s interactions with numerous ligands give it the capacity to both sense and respond to signals and provides a potential ‘meeting point’ for various thrombophilic stimuli to converge.

B2GPI may also play a regulatory role in important immune pathways that could in turn be disrupted by the presence of anti-B2GPI. For example, a recent study demonstrated that the elongated, membrane-bound form of B2GPI acts as a binding site for the complement protein, C3 [11]. The complex of B2GPI and C3 may then in turn serve a dual purpose. In addition to opsonising apoptotic cells, C3 binding to B2GPI provides a binding site for factor H which then mediates degradation of C3 via the activity of factor I. There are suggestions that complement dysregulation plays a part in APS as evidenced by data from mouse models of APS showing that inhibition of C3 activity can prevent fetal loss [12,13]. Furthermore, inhibition of complement component C5 by eculizumab has been used to treat patients with catastrophic APS [14]. However, how the complement pathway is affected by the presence of anti-B2GPI is yet to be determined.

## 3. Testing for Anti-B2GPI: An Evolving Component of the Laboratory Criteria for APS

The identification of B2GPI as a target for the pathogenic pathways of APS has prompted studies to look at the utility of anti-B2GPI in APS diagnosis. Consensus guidelines for APS were originally compiled in the 1990s and pertained to lupus anticoagulant (LA) and anticardiolipin antibodies (aCL) [15]. However, they were subsequently updated in 2006 on account of several studies indicating a role for anti-B2GPI to identify APS patients with both vascular and obstetric APS [3]. Estimates of the prevalence of anti-B2GPI in APS vary and this may be attributable to the heterogeneity of patient populations as well as differences in assays used. Several studies have reported isolated anti-B2GPI and this may account for between 11% and 27% of APS patients [16,17]. Isolated anti-B2GPI seems to occur more frequently than either isolated LA or isolated aCL; accounting for 75% of APS patients who had a single antiphospholipid antibody in one study [17]. Analysis of isolated anti-B2GPI has also been performed in the specific context of obstetric APS. Among 500 healthy women who were prospectively screened for aPL in early pregnancy, 4% were found to have anti-B2GPI without other aPL. Pre-eclampsia and eclampsia occurred significantly more frequently among these women compared with aPL-negative women raising a potentially important association with this particular obstetric complication [18]. A recent systematic review and meta-analysis of the risk of thrombotic events according to aPL type indicated that the association of anti-B2GPI is greater for arterial events than for venous thrombosis in patients without systemic lupus erythematosus (SLE) [19].

## 4. Significance of Anti-B2GPI Isotypes: An Ongoing Area of Contention

Following recognition of the importance of anti-B2GPI in the development of APS, further study has been directed towards understanding the significance of particular isotypes of anti-B2GPI. This issue was addressed at the International Congress on Antiphospholipid Antibodies in 2010 and an update to the international consensus guidelines on anticardiolipin and anti-B2GPI testing was published in 2012 [20]. These guidelines conclude that the evidence for an association between anti-B2GPI and APS is strongest for the IgG isotype. The guidelines acknowledge that data continues to build for IgA anti-B2GPI, but currently testing for this isotype is recommended in patients with negative IgG and IgM anti-B2GPI and in whom APS is still suspected. However, debate continues as new studies emerge that discuss the relative merits of the three anti-B2GPI isotypes.

### 4.1. Utility of IgM Anti-B2GPI

The value of IgM anti-B2GPI testing in APS has been supported by reports of patients with isolated IgM anti-B2GPI as the sole aPL. For example, in an Italian study of 64 primary APS patients (diagnosed on clinical grounds) with only one aPL detected, over 50% were positive for IgM anti-B2GPI. Approximately two-thirds of those with isolated IgM anti-B2GPI had obstetric manifestations of APS, perhaps indicating a possible association of IgM anti-B2GPI and this particular subtype of APS [17].

By contrast, several other studies have reported a stronger association between IgG anti-B2GPI and clinical manifestations of APS compared with IgM anti-B2GPI which has cast considerable doubt on the role of IgM anti-B2GPI in the first-line assessment of APS. In one of the first studies to examine this issue, Lakos *et al.* reported no association between IgM anti-B2GPI and typical APS manifestations, including venous thrombosis and miscarriage, in contrast to IgG anti-B2GPI [21]. Another study looked at the ability of anti-B2GPI isotypes to stratify thrombotic risk in patients with lupus anticoagulant, and found that those with IgG anti-B2GPI had a higher incidence of thrombosis whereas there was no association for IgM anti-B2GPI [22]. A recent study from Western Australia assessed the clinical phenotype of 128 hospital patients who had tested positive for at least one anti-B2GPI isotype [23]. There was a higher proportion of patients with a history of unprovoked venous and arterial thrombosis among those who were IgG anti-B2GPI positive compared to those who lacked IgG anti-B2GPI. This was not the case for IgM or IgA anti-B2GPI. Similarly, median IgG anti-B2GPI levels were higher among patients with unprovoked thrombosis compared to those with clinical events less in keeping with APS. The reverse trend was observed for IgM anti-B2GPI antibody levels suggesting that IgM anti-B2GPI had the least robust clinical association with potential APS. 

Indeed, some data suggests that IgM anti-B2GPI may actually play a protective role against disease in some situations. In a study of nearly 800 patients with SLE, those with IgM anti-B2GPI had a lower incidence of lupus nephritis, hypertension and renal impairment. Furthermore, IgM anti-B2GPI did not associate with arterial or venous thrombosis in contrast to IgG and IgA anti-B2GPI isotypes [24].

Given mounting evidence that IgM anti-B2GPI may not be strongly associated with APS, there is concern that its continued inclusion as a first line aPL test for APS is no longer warranted. Indeed the data currently suggest that IgM anti-B2GPI is less likely than other isotypes to associate with APS. However, a persistently positive IgM anti-B2GPI result could help substantiate a diagnosis of APS for individual patients presenting with thrombotic or obstetric complications. Therefore, further discussion regarding how to balance these conflicting ideas from the standpoint of the diagnostic laboratory is required to achieve a pragmatic testing strategy that is helpful to clinicians.

### 4.2. Associations of IgA Anti-B2GPI

Of the three anti-B2GPI isotypes, the role for IgA anti-B2GPI testing remains the most contentious and ambiguous. The first reports of IgA anti-B2GPI’s potential role in APS diagnosis emerged in the late 1990s from case reports of patients with APS and IgA anti-B2GPI with an otherwise negative aPL profile and also from relatively small patient cohort studies demonstrating an association between IgA anti-B2GPI and APS manifestations [21,25,26,27]. Since then, numerous other studies examining this area have been published, yet uncertainty remains regarding key questions such as the prevalence of IgA anti-B2GPI in health and disease. After nearly 20 years of study, the prevalence of IgA anti-B2GPI in primary and secondary APS has not yet been definitively established, with a variety of reported estimates ranging from 14% to 72% [28]. Methodological differences between studies such as differences in patient demographics and clinical phenotypes (e.g., patient ethnicities, primary *vs.* secondary APS, thrombotic *vs.* obstetric manifestations, *etc*.), varying assay methods and small patient numbers have added complexity to the interpretation of the data.

After initial reports of a positive association between IgA anti-B2GPI and APS manifestations, several studies published data disputing a role for this isotype in APS diagnosis. Danowski *et al.* reported that among a group of approximately 400 SLE patients with APS, IgA anti-B2GPI did not associate with any manifestation of APS in contrast to IgG and IgM anti-B2GPI [16]. Samarkos *et al.* reported similar findings among patients with primary APS and SLE [29].

Given the conflicting data regarding the utility of IgA anti-B2GPI testing in APS, consensus guidelines have not definitively included or excluded the isotype from testing algorithms. Rather, it is suggested that testing remains an option for individual patients who are negative for other aPL. This standpoint may be debated again when the guidelines are next reviewed in light of recent studies continuing to support IgA anti-B2GPI’s role in APS including from larger patient cohort studies.

Indeed, one of the largest studies to date examining the utility of IgA anti-B2GPI testing included nearly 6000 patients [30]. The majority of this group (5098 patients) were patients being assessed for potential APS at the Antiphospholipid Standardisation Laboratory in Texas. The remainder (approximately 900 patients) were drawn from two established cohorts of patients with SLE. The overall prevalence of IgA in the non-SLE group was low at <1% compared with approximately 20% in the SLE patient groups. Isolated IgA anti-B2GPI prevalence was <0.5% and 5% in the non-SLE and SLE groups respectively. A considerable number of patients with IgA anti-B2GPI had at least one APS-related clinical manifestation which included classical APS presentations as well as non-classical features such as thrombocytopenia and livedo reticularis. An interesting finding to emerge among the groups of patients with SLE was that IgA anti-B2GPI was significantly associated with arterial thrombosis but not venous thrombosis. A recent study from Spain also reported a stronger association with arterial thrombosis for IgA anti-B2GPI compared with IgG or IgM anti-B2GPI among 156 patients who met clinical criteria for APS [31]. This study also reported significantly different prevalence for IgA anti-B2GPI among patients with primary APS compared with APS associated with systemic autoimmune disease. These findings could conceivably indicate a variable role for IgA anti-B2GPI in different pathogenic pathways of APS resulting in distinct clinical phenotypes.

A major challenge to studying aPL and their associations with APS relates to the variability in diagnostic assays and lack of assay standardisation. This is particularly relevant to IgA anti-B2GPI which exhibits greater variability in results between assays compared to IgG and IgM isotypes. For example, a recent study compared results for IgA anti-B2GPI from approximately 70 patients using seven different commercial enzyme-linked immunosorbent assay (ELISA) kits and demonstrated substantial differences in sensitivity and specificity between assays [32]. Similar findings were also discussed in the Antiphospholipid Antibodies Task Force report on laboratory diagnostics pertaining to data from cohorts of APS and SLE patients. Comparisons of assays for each anti-B2GPI isotype identified a lower level of agreement between assays for IgA- compared with IgG- and IgM anti-B2GPI [33].

## 5. Anti-B2GPI Outside of APS: Questions Regarding Specificity

The precise role of anti-B2GPI isotypes in APS remains incompletely resolved and this can often lead to clinical uncertainty when interpreting the significance of a positive anti-B2GPI result. As discussed already, the prevalence of anti-B2GPI antibodies among patients with different diseases has not been firmly established but would provide important data when considering the specificity of anti-B2GPI antibodies. It is well known that anticardiolipin antibodies can be seen in conditions other than APS. Medications, infections and other illnesses have been reported in association with aCL which are often transient and unsustained [34]. Less is known about the associations and significance of anti-B2GPI outside of APS but several studies have addressed this issue in selected patient populations with diseases other than APS. For example, infections are thought to be an important trigger of aPL production and this phenomenon is thought to be due in part to molecular mimicry [34]. Indeed, a study of patients in South Africa found anti-B2GPI antibodies in 6-8% of patients of HIV, syphilis and malaria and in 89% and 30% respectively of patients with leprosy and hepatitis C [35].

With regard to disease associations of particular isotypes, an increased prevalence of IgA anti-B2GPI has been reported in a variety of disorders such as autoimmune hepatitis, coeliac disease, metabolic syndrome, and haemodialysed patients with end stage renal failure [36,37,38,39]. The significance of these associations remains unclear as the presence of anti-B2GPI is not always associated with APS manifestations. However, in some circumstances, the presence of IgA anti-B2GPI may confer a worse prognosis of the underlying disease. For example, in the case of end stage renal failure patients receiving haemodialysis, IgA anti-B2GPI were an independent risk factor for mortality and antibody levels fell in patients who received a renal transplant [39,40].

## 6. Obstetric APS and Anti-B2GPI

Obstetric APS represents a subset of APS and is thought to be mediated by distinct pathogenic mechanisms. As well as thrombosis of placental vessels, non-thrombotic mechanisms are also thought to be important. These include binding of anti-B2GPI to trophoblasts resulting in modulation of trophoblast proliferation and growth. Anti-B2GPI may also affect endometrial cells in the decidua that might impede implantation. Complement activation and enhanced apoptosis of embryonic and placental cells may also play a role in the pathways that lead to recurrent early miscarriage, fetal loss, pre-eclampsia and placental insufficiency [5]. Consequently, it has been hypothesised that patients with obstetric APS may differ in their aPL profiles compared with patients with predominantly vascular APS. As with vascular APS, it is difficult to combine evidence from obstetric APS studies on account of variability in study design, clinical case definition, range of aPL tested variance of laboratory reference ranges from consensus guideline recommendations and other constraints. This has been discussed in detail by the Antiphospholipid Antibody Task Force who reported their findings from the 14th International Congress on antiphospholipid antibodies [41].

The available data continue to show conflicting results regarding the utility of anti-B2GPI in obstetric APS. A meta-analysis of studies pertaining to placental problems in pregnancy (comprising late fetal loss, preeclampsia, placental abruption or intrauterine growth restriction), identified two cohort studies which demonstrated an association between anti-B2GPI and preeclampsia and late fetal loss [18,42,43]. One cohort study demonstrated an association with intrauterine growth restriction. However, there were four other case-control studies which did not confirm these associations [44,45,46,47]. The meta-analysis concluded that there was insufficient data to establish a significant link between anti-B2GPI and pregnancy morbidity. By contrast, LA was found to associate with placenta mediated complications with an odds ratio of approximately 10 for late fetal loss among cohort studies. More recently, a large multicentre prospective study including nearly 600 cases of fetal death after 20 weeks gestation found that elevated levels of IgM and IgG anti-B2GPI were associated with an increased risk of stillbirth, with odds ratios of 2 and 3 respectively [48]. One factor that has hampered direct comparison of various aPL in obstetric APS has been the heterogeneity of antibodies tested among different studies. A limited number of studies have included triple testing of LA, aCL and anti-B2GPI and indicate that positivity for all three antibodies is a risk factor for pregnancy morbidity [41,49].

The majority of studies in obstetric APS have included testing for IgG and/or IgM anti-B2GPI. However, there have been several studies that have examined the associations of IgA anti-B2GPI in relation to obstetric outcomes. Perhaps unsurprisingly, the data shows conflicting results. For example, a retrospective study in the early 2000s found that pregnant women with APS, preeclampsia and autoimmune disease had significantly higher IgA anti-B2GPI levels compared with a group of pregnant patients with diabetes and women with normal pregnancies [50]. Another study also found higher IgA anti-B2GPI levels among women with recurrent spontaneous miscarriages and fetal loss [51]. However, a study looking at anti-B2GPI antibodies in 84 women with primary APS, unexplained pregnancy morbidity and SLE found a low prevalence of IgA anti-B2GPI and no significant clinical associations [52].

Overall, the evidence relating to the significance of anti-B2GPI in obstetric APS is hindered by differences in the clinical and epidemiological characteristics of patients studied, as well as methodological differences in assays used. Longitudinal, prospective studies are needed in this field to help resolve the ambiguities regarding the prevalence and significance of anti-B2GPI in obstetric APS. 

## 7. Future Directions for Anti-B2GPI Testing

### 7.1. Refining the Target of Anti-B2GPI Testing: The Role of Domain Specific Antibodies

There are many difficulties in studying anti-B2GPI antibodies and one of the key remaining questions is how to identify patients with the highest risk of thrombosis or obstetric morbidity—*i.e.*, why do some patients with anti-B2GPI develop APS and others do not? A current area of assay development aims to distinguish between different subtypes of anti-B2GPI antibody that may differ in their pathogenicity. Standard anti-B2GPI assays do not differentiate autoantibodies against different domains of B2GPI. There is emerging evidence to suggest that determining the target domain of anti-B2GPI antibodies may improve the specificity and utility of testing.

A multicentre study of 447 patients assessed the significance of domain 1 specific IgG anti-B2GPI antibodies compared with IgG anti-B2GPI against other domains [53]. In this study, 55% of patients had IgG antibodies directed against domain 1 of B2GPI. Of these patients, 83% had a history of thrombosis. By contrast, among patients with IgG anti-B2GPI against other domains of B2GPI, a smaller proportion (58%) had a history of thrombosis. Furthermore, domain 1 specific anti-B2GPI showed a significant association with obstetric manifestations of APS in this patient group which was not observed for anti-B2GPI against other domains. 

Domain specific antibodies may also be helpful in refining the diagnostic value of IgA anti-B2GPI. A study looking at a small number of SLE patients with IgA anti-B2GPI found that domain 4 and 5 specific antibodies were seen more commonly among SLE patients without thrombosis compared to those with a history of thrombosis [54]. If verified in larger cohorts of patients, domain specific anti-B2GPI antibodies could potentially help stratify patients with a higher risk of APS in association with systemic autoimmune disease which might inform clinical decisions such as when to offer anticoagulant prophylaxis. However, domain specific anti-B2GPI testing is not yet widely available in most diagnostic laboratories.

### 7.2. Improved Standardisation of Anti-B2GPI Testing

There are numerous challenges relating to assays used for assessment of anti-B2GPI and these are discussed extensively elsewhere [55]. These include variation in isotypes tested by different centres, variability in the sensitivity and specificity of assays, significant inter-laboratory and intra-assay variability, the lack of universally agreed units of measurement and lack of reference material for traceability of measurements.

These issues are the focus for improving the diagnostic utility of anti-B2GPI testing in APS. One area in progress is the development of IgG and IgM reference material derived from pooled serum from well characterised APS patients with very high anti-B2GPI levels. The Antiphospholipid Antibodies Task Force recently reported that further validation studies were being performed and it is hoped that this will help to address issues regarding standardisation of assays and reduce inter-laboratory and inter-assay variability [33]. There are no reference materials for IgA anti-B2GPI, and this is an area for development if IgA anti-B2GPI is to be more widely adopted in anti-B2GPI testing algorithms.

## 8. Conclusions

B2GPI is a multifunctional protein with key roles in the clotting pathway. Antibodies against B2GPI contribute to APS and are particularly important in the subset of patients who test negative for other aPL. Current data support the inclusion of anti-B2GPI in the laboratory diagnostic criteria of APS and in particular, favour IgG anti-B2GPI over IgM in terms of specificity for APS. There is accumulating data to support a role for IgA anti-B2GPI in APS diagnosis, but its exact significance in relation to particular APS clinical phenotypes remains unresolved. The laboratory diagnosis of APS continues to be a challenging area of research as there is considerable variability in patient populations studied, assays used and the majority of studies have been retrospective. Coordination of prospective, longitudinal studies is vital to improve our understanding of this fascinating group of autoantibodies.

## Abbreviations

The following abbreviations are used in this manuscript:

APS	Antiphospholipid syndrome
B2GPI	Beta-2 glycoprotein I
Anti-B2GPI	Anti-beta-2 glycoprotein I antibodies
SLE	Systemic lupus erythematosus
aPL	Antiphospholipid antibody
LA	Lupus anticoagulant
aCL	Anticardiolipin
IgG	Immunoglobulin G
IgM	Immunoglobulin M
IgA	Immunoglobulin A
ELISA	Enzyme-linked immunosorbent assay
